# Guide RNAs with embedded barcodes boost CRISPR-pooled screens

**DOI:** 10.1186/s13059-019-1628-0

**Published:** 2019-01-24

**Authors:** Shiyou Zhu, Zhongzheng Cao, Zhiheng Liu, Yuan He, Yinan Wang, Pengfei Yuan, Wei Li, Feng Tian, Ying Bao, Wensheng Wei

**Affiliations:** 1 Biomedical Pioneering Innovation Center (BIOPIC), Beijing Advanced Innovation Center for Genomics, Peking-Tsinghua Center for Life Sciences, Peking University Genome Editing Research Center, State Key Laboratory of Protein and Plant Gene Research, School of Life Sciences, Peking University, Beijing, 100871 China; 2 Academy for Advanced Interdisciplinary Studies, Peking University, Beijing, 100871 China; 3 EdiGene Inc, Life Science Park, 22 KeXueYuan Road, Changping District, Beijing, 102206 China; 4 Center for Genetic Medicine Research, Children’s National Health System, Washington, DC, 20010 USA; 5 Department of Genomics and Precision Medicine, The George Washington School of Medicine and Health Sciences, Washington, DC, 20010 USA

## Abstract

We report a new method using re-designed guide RNAs with internal barcodes (iBARs) embedded in their loop regions. Our iBAR approach outperforms the conventional method by producing screening results with much lower false-positive and false-negative rates especially with a high multiplicity of infection (MOI). Importantly, the iBAR approach reduces the starting cells at high MOI significantly with greatly improved efficiency and accuracy compared with the canonical CRISPR screens at a low MOI. This new system is particularly useful when the source of cells is limited or when it is difficult to control viral infection for in vivo screening.

**Electronic supplementary material:**

The online version of this article (10.1186/s13059-019-1628-0) contains supplementary material, which is available to authorized users.

## Introduction

The CRISPR/Cas9 system enables editing at targeted sites in the genome with high efficiency and specificity [1–3]. One of its extensive applications is to identify the functions of coding genes, non-coding RNAs, and regulatory elements through high-throughput pooled screening in combination with next-generation sequencing (NGS) analysis. By introducing a pooled single-gRNA (sgRNA) or paired-gRNA (pgRNA) library into cells expressing Cas9 or catalytically inactive Cas9 (dCas9) fused with effector domains, investigators can perform multifarious genetic screens by generating diverse mutations, large genomic deletions, transcriptional activation, or transcriptional repression [4–11]. To generate a high-quality cell library of gRNAs for any given CRISPR screen, one must use a low multiplicity of infection (MOI) during cell library construction to ensure that the majority of cells harbors one sgRNA/pgRNA to minimize the false-positive discovery rate [7, 12, 13]. To further reduce the FDR (false discovery rate) and increase data reproducibility, in-depth coverage of gRNAs and multiple biological replicates are often necessary to obtain the hit genes with high statistical significance [12], resulting in increased library size. Additional difficulties may arise when one performs a large number of genome-wide screens, when the cell materials for library construction are limited, or when one conducts more challenging screens (i.e., in vivo) for which it is difficult to arrange the experimental replications or control the MOI. To address these technical difficulties, we aimed to design a novel method that enabled us to use a high MOI to generate CRISPR libraries in target cells and to obtain improved statistics for the screening results. We hypothesized that both the false-positive and false-negative rates of high MOI screens could be drastically reduced if we had more experimental replicates for each of the gRNAs. If we assigned the gRNAs different barcodes that were somehow embedded within the gRNA sequences, we could then trace the performance of each gRNA multiple times within the same experiment by counting both the gRNAs and their internal barcode (iBAR) sequences.

Two papers have recently reported methods to generate random barcodes outside the sgRNA body for pooled CRISPR screening [14, 15]. Assuming each sgRNA would create both desired loss-of-function (LOF) and non-LOF alleles, calculating all reads of any given sgRNA is unable to accurately assess the importance of its targeting gene in negative screening. Much improved statistical results could be achieved by linking one UMI (unique molecular identifier) with one editing outcome of each sgRNA to enable single-cell lineage tracing so as to lower the false-negative rate [14], or by counting the decreased number of RSLs (random sequence labels) affiliated with sgRNAs to improve screening quality [15]. Different from these two methods, we aimed to develop a method through embedded iBARs to enable pooled screening with CRISPR library made of viral infection at a high MOI, so as to reduce library size and improve data quality.

## Results

### A design-based CRISPR sgRNA^iBAR^ library screening method

**Fig. 1:**
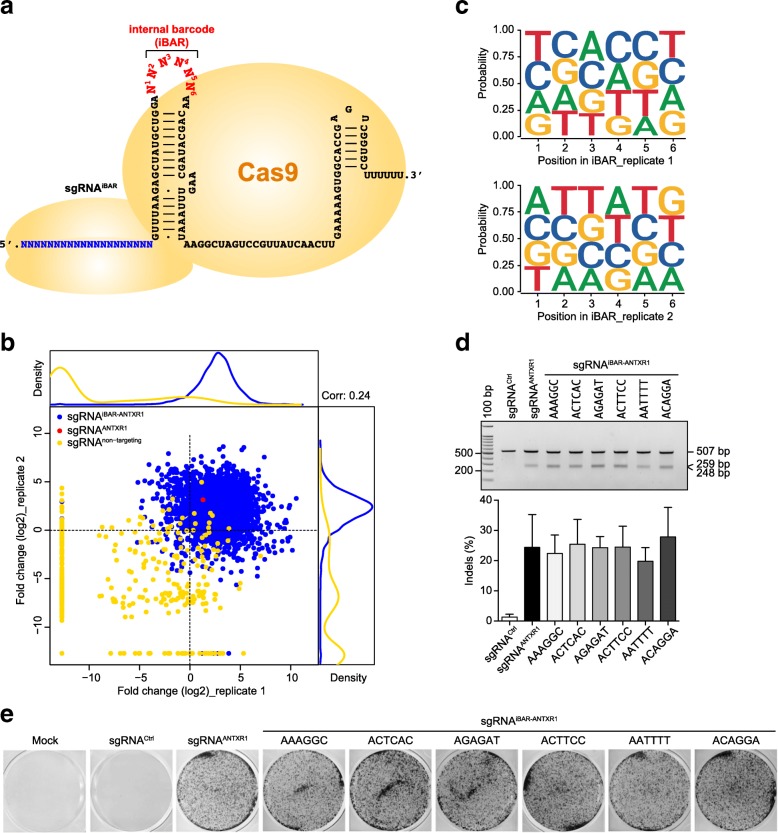
A design-based CRISPR screening via the gRNA^iBAR^ method. **a** Schematic diagram of an sgRNA with an internal barcode (iBAR). A 6-nt barcode (iBAR_6_) was embedded in the tetra loop of the sgRNA scaffold. **b** CRISPR screening of a collection of one particular sgRNA^ANTXR1^ containing all 4096 types of iBAR_6_. Fold changes between the reference and toxin treatment groups were calculated using the normalized abundances of sgRNAs^ANTXR1^. A density plot of barcodes and non-targeting sgRNAs is presented. Corr: Pearson correlation. **c** Effects of nucleotides at each position of the iBAR on fold changes of guide RNAs. **d** Indels generated by sgRNA^iBAR-ANTXR1^ associated with six barcodes that appeared to be the worst in conferring cell resistance to PA/LFnDTA from the above screening. Percentages of cleavage efficiency in the T7E1 assay were measured using Image Lab software, and data are presented as the mean ± s.d. (*n* = 3). All primers used are listed in Additional file 3: Table S9. **e** MTT viability assay for the effects of the indicated sgRNA^iBAR-ANTXR1^ on the susceptibility of cells to PA/LFnDTA

Ideally, the embedded iBAR should not affect the efficiency of the gRNA in guiding the Cas9 or dCas9 nuclease to the target site. We decided to place the barcode sequence in the tetra loop of the gRNA scaffold outside of the Cas9-sgRNA ribonucleoprotein complex, which has been subjected to frequent alterations for various purposes maintaining enough activity of its upstream guide sequence [11, 16]. We arbitrarily designed a 6-nt-long iBAR (iBAR_6_) that gave rise to 4096 barcode combinations, providing sufficient variation for our purposes (Fig. 1a). To determine whether the insertions of these extra iBAR sequences affected the gRNA activities, we constructed a library of a pre-determined sgRNA targeting the anthrax toxin receptor gene *ANTXR1* [17] in combination with all 4096 types of iBAR_6_. This special sgRNA^iBAR-ANTXR1^ library was constructed in HeLa cells that constantly express Cas9 [7, 8] through lentiviral transduction at a low MOI of 0.3. After three rounds of PA/LFnDTA toxin treatment and enrichment, the sgRNA along with its iBAR_6_ sequences from toxin-resistant cells were examined through NGS analysis as previously reported [7]. The majority of sgRNAs^iBAR-ANTXR1^ and the sgRNAs^ANTXR1^ without barcodes were significantly enriched, whereas almost all the non-targeting control sgRNAs were absent in the resistant cell populations. Importantly, the enrichment levels of sgRNAs^iBAR-ANTXR1^ with different iBAR_6_s appeared to be random between two biological replicates (Fig. 1b). After calculating the nucleotide frequency at each position of iBAR_6_, we failed to observe any bias of nucleotides from either of the replicates (Fig. 1c). Additionally, the GC contents in iBAR_6_ did not seem to affect the sgRNA cutting efficiency (Additional file 1: Figure S1). However, there was a small number of iBAR_6_s whose affiliated sgRNA^ANTXR1^ did not perform well in either screening replicate (Additional file 2: Table S1). To rule out the possibility that these iBAR_6_s had negative effects on sgRNA activity, we selected six different iBARs from the bottom of the sgRNA^iBAR-ANTXR1^ ranking for further investigation. Compared to the control sgRNA^ANTXR1^ without a barcode, all six of these sgRNAs^iBAR-ANTXR1^ showed comparable efficiency in generating both DNA double-stranded breaks (DSBs) at target sites (Fig. 1d) and *ANTXR1* gene disruption leading to the toxin resistance phenotype (Fig. 1e). We further confirmed the negligible effects of iBARs on sgRNA efficiency by four different sgRNAs targeting *CSPG4*, *MLH1*, and *MSH2*, respectively (Additional file 1: Figure S2). Taken together, these results indicate that this re-designed sgRNA^iBAR^ retains sufficient activity of sgRNA, making it possible to generally apply this strategy in CRISPR-pooled screens.

**Fig. 2:**
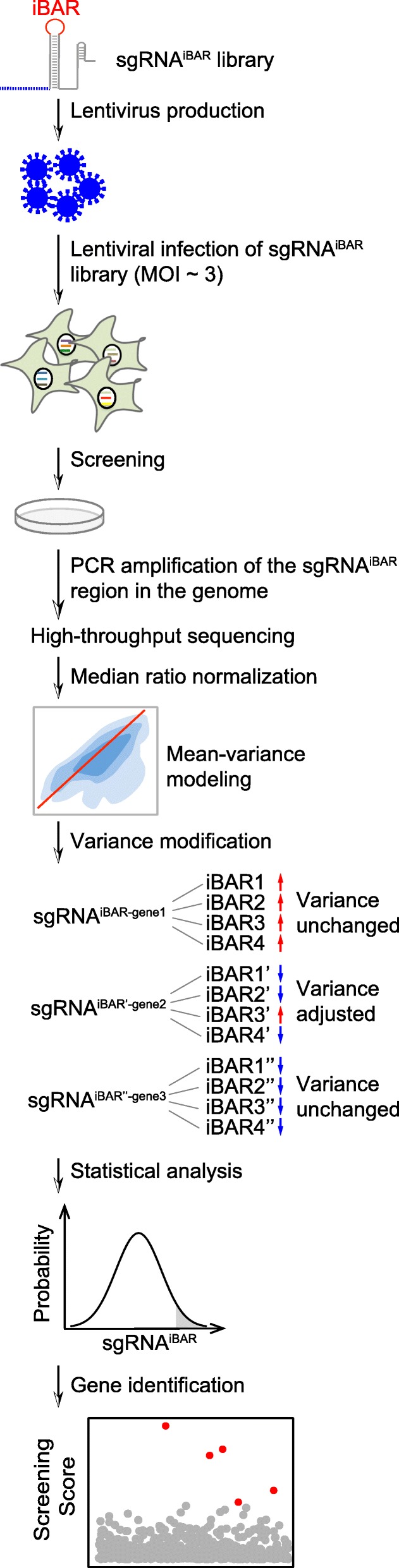
Schematic of CRISPR-pooled screening using sgRNA^iBAR^. For a given sgRNA^iBAR^ library, four different iBAR_6_s were randomly assigned to each sgRNA. The sgRNA^iBAR^ library was introduced into target cells through lentiviral infection with a high MOI (i.e., ~ 3). After library screening, sgRNAs with their associated iBARs from enriched cells were determined through NGS. For data analysis, median ratio normalization was applied, followed by mean-variance modeling. The variance of sgRNA^iBAR^ was determined based on the fold change consistency of all iBARs assigned to the same sgRNA. The *P* value of each sgRNA^iBAR^ was calculated using the mean and modified variance. Robust rank aggregation (RRA) scores of all genes were considered to identify hit genes. A lower RRA score corresponded to a stronger enrichment of the hit genes

Based on the iBAR strategy, we then set out to broaden its application to perform a novel sgRNA^iBAR^ library screen at a high MOI. We followed the standard procedure to harvest the library cells, extract their genomic DNA for PCR amplification of sgRNA with iBAR coding regions, and perform NGS analysis [7, 12, 13]. The MAGeCK algorithm could be used to calculate the statistical significance of an sgRNA score through normalization of its raw counts, estimation of its variance using a negative binomial (NB) model, and determination of its ranking using a null model with a uniform distribution [18]. Taking the iBAR into consideration, we assessed the consistency of any sgRNA count change among all the associated iBARs within the same experimental replicate. This process effectively eliminates free riders that were associated with functional sgRNAs due to lentiviral infection at a high MOI in cell library construction. Specifically, for the iBAR system, we purposely adjusted the model-estimated variance for only those sgRNAs whose fold changes with multiple iBARs were in opposite directions, resulting in increased *P* values for these outliers. Finally, we identified hit genes based on sgRNA scores and technical variance between biological replicates (Fig. 2 and “Methods” section). We developed this specific MAGeCK-based algorithm named MAGeCK^iBAR^ for the analysis of gRNA^iBAR^ library screening that is open source and freely available for download (“Methods” section).

### Comparison of screenings at MOI of 0.3, 3, and 10 for essential genes involved in TcdB toxicity

We then constructed an sgRNA^iBAR^ library covering every annotated human gene. For each of the 19,210 human genes, three unique sgRNAs were designed using our newly developed DeepRank method (“Methods” section), each of which was randomly assigned four iBAR_6_s. In addition, 1000 non-targeting sgRNAs, each with four iBAR_6_s, were included as negative controls (Additional file 2: Table S2). For the ease of statistical comparison, every set of three unique non-targeting sgRNAs was artificially named a negative control gene. The 85-nt sgRNA^iBAR^ oligos were designed in silico (Additional file 1: Figure S3), synthesized using array synthesis, and cloned as a pooled library into a lentiviral backbone. Cas9-expressing HeLa cells were transduced with the sgRNA^iBAR^ library lentivirus at three different MOIs (0.3, 3, and 10) with 400-fold coverage for sgRNAs to generate cell libraries, in which each sgRNA^iBAR^ was covered 100-fold. To evaluate the effect of iBAR design for CRISPR screening at different MOIs, we performed a positive screening to identify genes that mediate the cytotoxicity of *Clostridium difficile* toxin B (TcdB), one of the key virulence factors of this anaerobic bacillus [19]. We have previously reported the first identification of the functional receptor of TcdB, CSPG4 [20], whose coding gene was also identified and ranked at the very top from a genome-scale CRISPR library screening [21]. In this reported CRISPR screening, *UGP2* gene was also top-ranked hit, and *FZD2* was identified and confirmed to encode the secondary receptor that mediates the TcdB’s killing effect on host cells. Of note, the role of FZD2 was significantly dwarfed by CSPG4 so that the *FZD2* gene could only be identified using the truncated TcdB that had CSPG4-interacting region deleted [21]. In our screens on TcdB, we used MAGeCK^iBAR^ (Additional file 2: Table S3) and MAGeCK (Additional file 3: Table S4) to analyze data from iBAR and the conventional CRISPR screens, respectively. We consequently obtained top-ranked genes (FDR < 0.15) from both.

**Fig. 3:**
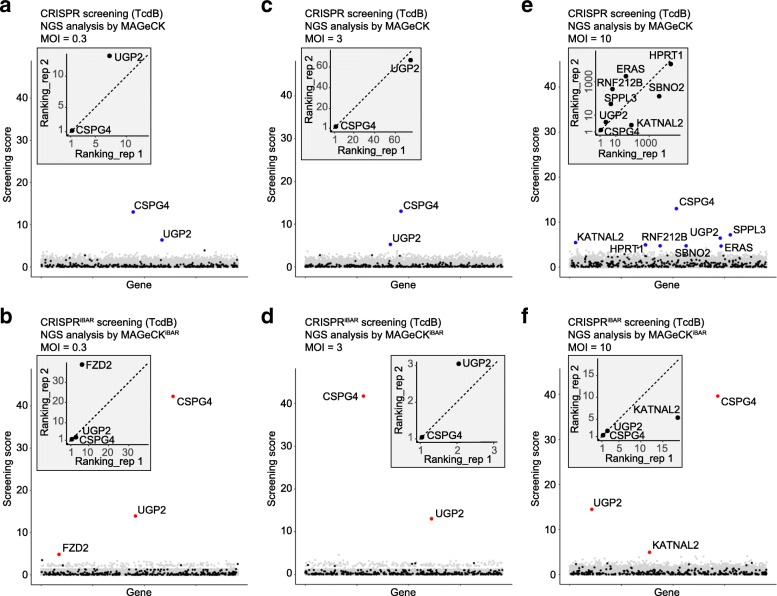
Screenings for essential genes involved in TcdB toxicity at MOI of 0.3, 3, and 10 in HeLa cells. **a**, **b** Screening scores of identified genes (FDR < 0.15) calculated by MAGeCK (**a**) and by MAGeCK^iBAR^ (**b**) at MOI of 0.3. **c**, **d** Screening scores of identified genes (FDR < 0.15) calculated by MAGeCK (**c**) and by MAGeCK^iBAR^ (**d**) at MOI of 3. **e**, **f** Screening scores of identified genes (FDR < 0.15) calculated by MAGeCK (**e**) and by MAGeCK^iBAR^ (**f**) at MOI of 10. Negative control genes are labeled with black dots. Rankings of identified candidates in each biological replicate through MAGeCK and MAGeCK^iBAR^ were presented

For screening at a low MOI of 0.3, *CSPG4* and *UGP2* were identified and ranked at the top (Fig. 3a), consistent with the previous report [21]. When taking iBARs into account, we identified *FZD2* in addition to *CSPG4* and *UGP2* (Fig. 3b). Because FZD2 is a proven receptor of TcdB which plays much weaker role than CSPG4 in HeLa cells [21], these results demonstrated that iBAR method offered superior quality and sensitivity to conventional CRISPR screening when constructing cell library at a low MOI. In addition, rankings of *CSPG4* and *UGP2* were far more consistent in CRISPR^iBAR^ screening between two experimental replicates, again indicating the much higher quality for the new method (Fig. 3a, b). At high MOIs (3 and 10), *CSPG4* and *UGP2* could be isolated from both CRISPR and CRISPR^iBAR^ screens, but the data quality was significantly higher with the latter (Fig. 3c–f). In general, the higher the MOI, the worse the signal-to-noise rate for the traditional method. At a MOI of 10, the number of false-positive hits was drastically increased in the conventional method, but not in CRISPR^iBAR^ screening (Fig. 3e, f). Impressively, *CSPG4* and *UGP2* remained top ranked from CRISPR^iBAR^ screening even at an MOI of 10, although the data quality slightly declined (Fig. 3f). Noticeably, nearly all *CSPG4*- and *UGP2*-targeting sgRNAs^iBAR^ were significantly enriched after TcdB treatment (Additional file 1: Figure S4), strikingly different from other genes identified at an MOI of 10 using conventional method, such as *SPPL3*, a likely false-positive result (Additional file 1: Figure S4). In comparison of the two biological replicates, *CSPG4* and *UGP2* were all ranked at the top in both biological replicates from CRISPR^iBAR^ screens with all MOI conditions (Fig. 3b, d, f), but not from the conventional CRISPR screens where *UGP2* was ranked lower than 60th in both replicates at an MOI of 3 (Fig. 3c) and many false-positive hits appeared in both replicates at an MOI of 10 (Fig. 3e). These results showed that iBAR method maintained the quality of data even at a high MOI as that at a low MOI for conventional CRISPR screening. Additionally, iBAR approach enables highly repeatable results between biological replicates as multiple replications could be conducted within one experiment (Fig. 3).

### Identification of genes important for 6-TG-mediated cytotoxicity using the CRISPR^iBAR^ and conventional CRISPR-pooled screens

To further evaluate the power of iBAR method, we went on conducting a screening to identify genes that modulate cellular susceptibility to 6-TG [22], a cancer drug that could be processed to inhibit DNA synthesis. We decided to construct the genome-scale sgRNA^iBAR^ library at a MOI of 3 to generate a cell library with high coverage (2000-fold) for each sgRNA, in which each sgRNA^iBAR^ was covered 500-fold. The overall read distribution of both experimental replicates was shown (Additional file 1: Figure S5a), and the reference cell libraries of both replicates reached 97% coverage of all originally designed sgRNAs (Additional file 1: Figure S5b). Over 95% of the sgRNAs in the original libraries retained three to four iBARs, indicating the good quality of libraries in which most sgRNAs had sufficient barcode variants for screening and data analysis (Additional file 1: Figure S5c). The fold change of all genes correlated well between the two biological replicates (Additional file 1: Figure S6). For the same 6-TG screening of two sgRNA library replicates, we also employed MAGeCK and MAGeCK^iBAR^ analyses. For MAGeCK^iBAR^, we consequently obtained adjusted variance and mean distributions for all the sgRNAs^iBAR^ that heightened the variance of sgRNAs with enrichment inconsistent among different iBAR repeats (Additional file 1: Figure S7).

**Fig. 4:**
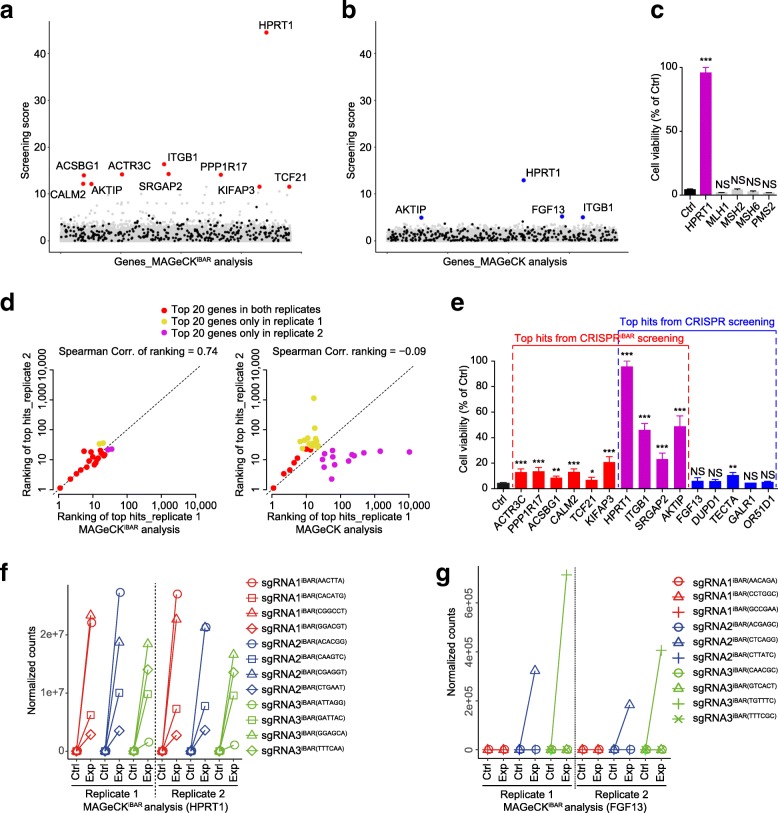
Comparison of the CRISPR^iBAR^ and conventional CRISPR-pooled screens for the identification of human genes important for 6-TG-mediated cytotoxicity in HeLa cells. **a**, **b** Screening scores of the top-ranked genes calculated by MAGeCK^iBAR^ (**a**) and by MAGeCK (**b**). Identified candidates (FDR < 0.15) were labeled, and only top 10 hits were labeled for MAGeCK^iBAR^ screens. Negative control genes were labeled with black dots. **c** Validation of reported genes (*MLH1*, *MSH2*, *MSH6*, and *PMS2*) involved in 6-TG cytotoxicity. **d** Spearman correlation coefficient of the top 20 positively selected genes between two biological replicates using MAGeCK^iBAR^ (left) or conventional MAGeCK analysis (right). **e** Validation of top candidate genes isolated by either MAGeCK^iBAR^ or MAGeCK analysis. Mini-pooled sgRNAs targeting each gene were delivered to cells through lentiviral infection. Transduced cells were cultured for an additional 10 days before 6-TG treatment. Data are presented as the mean ± S.E.M. (*n* = 5). *P* values were calculated using Student’s *t* test. *P < 0.05; **P < 0.01; ***P < 0.001; NS, not significant. The sgRNA sequences for validation are listed in Additional file 3: Table. S8. **f**, **g** The sgRNA^iBAR^ read counts for *HPRT1* targeting (**f**) and *FGF13* targeting (**g**) before (Ctrl) and after (Exp) 6-TG screening in two replicates

From the positively selected sgRNAs with statistical significance, we identified the top-ranked genes (FDR < 0.15) whose corresponding sgRNAs were consistently enriched among different iBARs (Fig. 4a, Additional file 3: Table S5), and we also found these top genes using the MAGeCK algorithm without taking barcodes into account (Fig. 4b, Additional file 3: Table S6). Consistent with a previous report [23], the sgRNAs targeting *HPRT1* gene were top ranked by both methods. Four genes (*MLH1*, *MSH2*, *MSH6*, and *PMS2*) were previously reported to be involved in 6-TG-mediated cell death [6]. We examined and confirmed the cutting activities of all except one of the primary designed sgRNAs targeting these four genes (Additional file 1: Figure S8), indicating that these genes were indeed irrelevant to 6-TG-mediated cell death in HeLa cells we used (Fig. 4c). When analyzing the two biological replicates separately, the top 20 genes of each replicate showed a high level of consistency with CRISPR^iBAR^ screening (Spearman correlation coefficient for rankings = 0.74), whereas the two replicates shared much less commonality when using the conventional method (Spearman correlation coefficient for rankings = − 0.09) (Fig. 4d, Additional file 3: Table S7).

To validate the screening results, we de novo designed and combined two sgRNAs to make a mini-pool to target each candidate gene, and each pool was introduced into HeLa cells through lentiviral infection (Additional file 3: Table S8). The effects of the sgRNA pools on cell viability against 6-TG treatment were quantified by a 3-(4,5-dimethyl-2-thiazolyl)-2,5-diphenyl-2-H-tetrazolium bromide (MTT) assay. Top 10 genes from CRISPR^iBAR^ as well as CRISPR screens were chosen for validation. Noticeably, two non-targeting control genes were identified and ranked in the top 10 candidate list from the conventional CRISPR screen (Additional file 3: Table S6). These evident false-positive results are predictable because of the high MOI we used to generate the cell library. We successfully confirmed that the top 10 candidate genes from CRISPR^iBAR^ of both replicates were all true-positive results; in contrast, only five genes from the top 10 candidate list from the conventional method turned out to be true positives (Fig. 4e). Among them, four genes (*HPRT1*, *ITGB1*, *SRGAP2*, and *AKTIP*) were obtained using both methods, whereas six genes (*ACTR3C*, *PPP1R17*, *ACSBG1*, *CALM2*, *TCF21*, and *KIFAP3*) were only identified and ranked at the top from CRISPR^iBAR^. In summary, iBAR improved accuracy with lower false-positive and false-negative rates for high MOI screens compared with conventional method.

We further assessed the performance of each sgRNA^iBAR^ targeting the top four candidate genes (*HPRT1*, *ITGB1*, *SRGAP2*, and *AKTIP*). All the different iBARs of the enriched sgRNAs appeared to have little effect on the enrichment levels of their affiliated sgRNAs, and the order of iBARs associated with any particular sgRNA appeared to be random (Additional file 1: Figure S9), further supporting our prior notion that the iBARs did not affect the efficiency of their affiliated sgRNAs. All four *HPRT1*-targeting sgRNAs^iBAR^ were significantly enriched after 6-TG treatment in both replicates (Fig. 4f). Most sgRNAs^iBAR^ of other CRISPR^iBAR^ identified genes were enriched after 6-TG selection (Additional file 1: Figure S10). In contrast, only a very few of sgRNAs^iBAR^ of some top-ranked genes from conventional CRISPR screening were enriched, including *FGF13* (Fig. 4g), *GALR1*, and two negative control genes (Additional file 1: Figure S11), leading to false-positive hits in the MAGeCK but not MAGeCK^iBAR^ analysis (Additional file 1: Figure S12).

## Discussion

The library coverage was significantly increased with a high MOI in the transduction with a fixed number of cells for library construction, so the starting cells for library construction could be decreased more than 10-fold (MOI = 3) and 35-fold (MOI = 10) to match and even top the results from conventional screening at an MOI of 0.3 (Additional file 3: Table S10). Four barcodes for each sgRNA, as we designed, appeared to provide sufficient internal repeats to enable the high level of consistency between the two biological replicates using the iBAR method (Fig. 3, Fig. 4d, Additional file 3: Table S7). Therefore, in addition to the significant reduction in cells for library construction, the internal replicates offered by iBARs within the same experiment would lead to more uniform conditions and fairer comparisons versus separate biological replicates, consequently improving statistical scores. The advantage of the iBAR method would become greater when large-scale CRISPR screens in multiple cell lines are in demand or when the cell samples for screening are scarce (i.e., samples from patients or those of primary origin). Especially for in vivo screening in which the lentiviral transduction rate is hard to predict and variable conditions in different animals might greatly impact the screening outcomes, the iBAR method could be an ideal solution to resolve these technical limitations.

Because multiple cuttings decrease cell viability, CRISPR library constructed at a high MOI might have abnormal false discovery rate for negative screening [24, 25]. We therefore performed a genome-scale negative screening at an MOI of 0.3 to assess the iBAR method in calling essential genes. For positive screening using iBAR, we modified the model-estimated variance of sgRNAs with different fold change directions among barcodes to enlarge variance so that the mis-associated sgRNAs were subjected to adequate penalty. For negative screening, however, sgRNA depletion through mis-association had little effect on its consistency of fold change directions as non-functional sgRNAs remained unchanged. Therefore, we treated barcodes only as internal replicates without the penalty procedure. We indeed achieved improved statistics with higher true-positive and lower false-positive rates for negative screening using iBAR method at a low MOI than the conventional approach using gold standard essential genes [26] (Additional file 1: Figure S13).

Notwithstanding the technical advancement of the iBAR method to offer the same benefit of internal replications, we must be cautious with the MOI during viral transduction to generate the original cell library in negative screens based on measuring cell viability. Although massive integrates have been reported not to affect cell fitness [27], multiple cuttings on DNA caused by higher MOI in cells with active Cas9 have been shown to reduce cell viability [24, 25]. Strategies without cuttings, such as CRISPRi/a [10] or iSTOP systems [28], could be better choices to combine with the iBAR system for negative screening at a high MOI.

Although we had data to support that iBAR_6_ had little effect on the activities of sgRNAs, we would not recommend to use barcodes with consecutive T (> 4) so as to avoid any minor effects. Ultimately, 4096 types of iBAR_6_ provided sufficient varieties to make CRISPR libraries. In addition, the length of the iBAR is not limited to 6 nt. We have tested different lengths of iBARs and found that their lengths could be up to 50 nt without affecting functions of their affiliated sgRNAs.

(Additional file 1: Figure S14). In addition, it is not necessary to design different barcode sets for different sgRNAs. A fixed set of iBARs assigned to all sgRNAs should work as well as random assignment in library screening. Our iBAR strategy with a streamlined analytic tool MAGeCK^iBAR^ would facilitate large-scale CRISPR screens for broad biomedical discoveries in various settings.

## Methods

### Cells and reagents

HeLa and HEK293T cell lines were maintained in Dulbecco’s modified Eagle’s medium (DMEM; Gibco C11995500BT) supplemented with 1% penicillin/streptomycin and 10% fetal bovine serum (FBS; CellMax BL102-02) and cultured with 5% CO_2_ at 37 °C. All cells were checked for the absence of mycoplasma contamination.

### Plasmid construction

The lentiviral sgRNA^iBAR^-expressing backbone was constructed by changing the position of the BsmBI (Thermo Scientific™, ER0451) site using BstBI (NEB, R0519) and XhoI (NEB, R0146) from Plenti-sgRNA-Lib (Addgene, #53121). sgRNA- and sgRNA^iBAR^-expressing sequences were cloned into the backbone using the BsmBI-mediated Golden Gate cloning strategy [29].

### Design of the genome-scale CRISPR sgRNA^iBAR^ library

Gene annotations were retrieved from the UCSC hg38 genome, which contains 19,210 genes. For each gene, three different sgRNAs that had at least one mismatch in the 16-bp seed region in the genome with a high level of predicted targeting efficiency were designed using our newly developed DeepRank algorithm. We then randomly assigned four 6-bp iBARs (iBAR_6_s) to each sgRNA. We designed an additional 1000 non-targeting sgRNAs, each with four iBAR_6_s, to serve as negative controls.

### Construction of the CRISPR sgRNA^iBAR^ plasmid library

The 85-nt DNA oligonucleotides were designed and array synthesized. Primers (oligo-F and oligo-R) targeting the flanking sequences of oligos were used for PCR amplification. The PCR products were cloned into the lentiviral vector constructed above using the Golden Gate method [29]. The ligation mixtures were transformed into Trans1-T1 competent cells (Transgene, CD501-03) to obtain library plasmids. Transformed clones were counted to ensure at least 100-fold coverage for the scale of the sgRNA^iBAR^ library. The library plasmids were extracted following the standard protocol (QIAGEN 12362) and transfected into HEK293T cells with the two lentivirus package plasmids pVSVG and pR8.74 (Addgene, Inc.) to obtain the library virus. The iBAR library containing all 4096 iBAR_6_s for one *ANTXR1*-targeting sgRNA was constructed using the same protocol.

### Screening of the sgRNA^iBAR-ANTXR1^ library containing all 4096 types of iBAR_6_

A total of 2 × 10^7^ cells were plated on 150-mm Petri dishes and infected with the library lentivirus at an MOI of 0.3. After 72 h of infection, cells were re-seeded and treated with 1 μg/ml of puromycin (Solarbio P8230) for 48 h. For each replicate, 5 × 10^6^ cells were collected for genome extraction. Screening of the sgRNA^iBAR-ANTXR1^ library was performed using PA/LFnDTA toxin [30, 31] after library-infected cells were cultured for 15 days [7]. Then, sgRNA with the iBAR coding region in genomic DNA was amplified (TransGen, AP131-13) using Primer-F and Primer-R and then subjected to high-throughput sequencing analysis (Illumina HiSeq2500) using an NEBNext Ultra DNA Library Prep Kit for Illumina (NEB E7370L).

### Screening of the genome-scale CRISPR/Cas9 sgRNA^iBAR^ library for genes important for TcdB cytotoxicity and for genes essential for cell viability

A total of 1.6 × 10^8^ cells (MOI = 0.3), 1.53 × 10^7^ cells (MOI = 3), and 4.6 × 10^6^ cells (MOI = 10) were plated on 150-mm Petri dishes respectively for sgRNA library construction for two replicates. Cells were infected with the library lentivirus of different MOIs and treated with 1 μg/ml of puromycin for 72 h post infection. sgRNA^iBAR^-integrated cells were cultured for an additional 15 days to maximize gene knockout. Cells were re-seeded onto 150-mm Petri dishes, treated by TcdB (100 pg/ml) for 10 h, and followed by the removal of the loosely attached round cells through repeated pipetting [20]. For each round of screening, the cells were cultured in fresh medium without TcdB to reach ~ 50–60% confluence. All resistant cells in one replicate were pooled and subjected to another round of TcdB screening. For the subsequent three rounds of screening, the TcdB concentration was 125 pg/ml, 150 pg/ml, and 175 pg/ml, respectively. After four rounds of treatment, the resistant cells and untreated cells were collected for genomic DNA extraction, amplification of sgRNA, and NGS analysis. Seven pairs of primers were used for PCR amplification (Additional file 3: Table S9), and PCR products were mixed for NGS. For negative screening at an MOI of 0.3, a total of 4.6 × 10^7^ (two replicates) sgRNA^iBAR^-integrated cells were cultured for 28 days before NGS decoding.

### Screening of the genome-scale CRISPR/Cas9 sgRNA^iBAR^ library for genes important for 6-TG cytotoxicity

A total of 5 × 10^7^ cells were plated on 150-mm Petri dishes, and two replicates were obtained. Cells were infected with the library lentivirus at an MOI of 3 and treated with 1 μg/ml puromycin 72 h after infection. sgRNA^iBAR^-integrated cells were cultured for an additional 15 days, re-seeded at a total number of 5 × 10^7^, and then treated with 200 ng/ml 6-TG (Selleck). For the following two rounds of screening, the 6-TG concentration was 250 ng/ml and 300 ng/ml. For each round of selection, the drug was maintained for 7 days, and the cells were cultured in fresh medium without 6-TG for another 3 days. Then, all the resistant cells in one replicate were grouped together and subjected to another round of 6-TG screening. After three rounds of treatment, the resistant cells and untreated cells were collected for genomic DNA extraction, amplification of sgRNA with iBAR regions, and deep-sequencing analysis.

### Positive screening data analysis

MAGeCK^iBAR^ is the analysis strategy developed for screens using an sgRNA^iBAR^ library based on the MAGeCK algorithm [18]. MAGeCK^iBAR^ takes great advantage of Python, Pandas, NumPy, and SciPy. The analysis algorithm contains three main parts: analysis preparation, statistical tests, and rank aggregation. In the analysis preparation stage, the inputted raw counts of sgRNAs^iBAR^ are normalized, and the coefficients of the population mean and variance are then modeled. In the statistical test stage, we use tests to determine the significance of the difference between the treatment and control normalized reads. In the rank aggregation stage, we aggregate the ranks of all the sgRNAs^iBAR^ targeting each gene to obtain the final gene ranking.

#### Normalization and preparation

We first obtained the raw counts of sgRNAs^iBAR^ from sequencing data. Because the sequencing depth and sequencing error might affect the raw counts of the sgRNAs^iBAR^, normalization was needed before the following analysis. A size factor was estimated to normalize the raw counts with different sequencing depths. However, because a few highly enriched sgRNAs might have strong influences on the total read counts, the ratio to total read counts should not be used in the normalization. Thus, we chose the median ratio normalization [32]. Suppose there were *n* sgRNAs in the library, with *i* ranging from 1 to *n*, and *m* experiments in total (both control and treatment groups), with *j* ranging from 1 to *m*. The size factor *s*_*j*_ can be expressed as follows:

$$ {s}_j=\mathrm{median}\left(\frac{k_{ij}}{\prod_{v=1}^m{k_{iv}}^{1/m}}\right) $$

Thus, we obtained the normalized counts of sgRNAs^iBAR^ in each experiment by calculating the corresponding size factor. In the mean-variance modeling step, the NB distribution was used to estimate the mean and variance of every sgRNA^iBAR^ across biological replicates and different treatments [33]:

$$ {K}_{ij}\sim NB\left({\mu}_{ij},{\sigma_{ij}}^2\right) $$

We used the model adopted by MAGeCK to calculate the coefficients of the mean and variance [18]. The mean-variance model satisfied the following relationship:

$$ {\sigma}^2=\mu +k{\mu}^b $$

To determine the *k* and *b* coefficients from all the sgRNAs^iBAR^ in the library, the function can be transformed into a linear function:

$$ {\log}_2\left({\sigma}^2-\mu \right)={\log}_2k+b{\log}_2\mu $$

The means of the treatment and control counts were calculated directly, and the corresponding variance could be calculated from the mean and coefficients. For the CRISPR-iBAR analysis, we evaluated the enrichment of sgRNAs through the performances of different iBARs. We designed four iBARs for each sgRNA to serve as internal replicates. Due to the high MOI during library construction, there must be free riders of false-positive sgRNAs associated with true-positive hits. The free rider here was used to describe the sgRNAs targeting irrelevant genes that were mis-associated with functional sgRNAs to enter the same cells. We modified the variance of sgRNAs^iBAR^ based on the enrichment directions of different iBARs for each sgRNA. If all the iBARs of one sgRNA presented the same direction of fold change, i.e., all greater or less than that of the control group, then the variance would be unchanged. However, if one sgRNA with different iBARs revealed inconsistent directions of fold change, then this kind of sgRNA would be penalized by increasing its variance. The final adjusted variance for inconsistent sgRNAs^iBAR^ would be the model-estimated variance plus the experimental variance calculated from the Ctrl and Exp samples.

Finally, the score of an sgRNA^iBAR^ was calculated by the mean and normalized variance of the treatment compared to those of the control group:

$$ {\mathrm{score}}_i=\frac{t_i-{c}_i}{v_i} $$

where *t*_*i*_ is the mean of the treatment counts of the *i*th sgRNA and *c*_*i*_ and *v*_*i*_ are the mean and variance of control counts of the *i*th sgRNA. Because the variance is used as the denominator to calculate the score, the enlarged variance for the inconsistent sgRNAs^iBAR^ results in lower score.

#### Statistical test and rank aggregation

The normal distribution was used to test the score_*i*_ of the treatment counts. The two sides of scores in a standard normal distribution provided the greater-tail and lesser-tail *P* value separately. To obtain the gene ranks, we used RRA, which is an appropriate method for aggregating rankings [34]. MAGeCK adopted a modified RRA method by limiting the enriched sgRNAs [18]. Suppose for one gene there are *n* sgRNAs with different iBARs in the library of M sgRNAs^iBAR^ in total; every sgRNA^iBAR^ has a rank in the library of *R* = (*R*_1_, *R*_2_,  … , *R*_*n*_). First, the ranks of sgRNAs^iBAR^ should be normalized by the total number of sgRNAs^iBAR^ in the library. We obtained the normalized rank *r* = (*r*, *r*_2_,  … , *r*_*n*_) for each *r*_*i*_ = *R*_*i*_/*M*, in which 1 ≤ *i* ≤ *n*. Then, we calculated the sorted normalized ranking *sr*, making *sr*_1_ ≤ *sr*_2_ ≤  …  ≤ *sr*_*n*_. The sorted normalized rank follows a uniform distribution between 0 and 1. The probability *β*_*k*, *n*_(*sr*) in which *sr*_*i*_ ≤ *r*_*i*_ follows a *β* distribution *β*(*k*, *n* + 1 − *k*), making *ρ* = min(*β*_1, *n*_, *β*_2, *n*_,  … , *β*_*n*, *n*_). For every gene, the *ρ* score can be obtained by RRA and further adjusted by Bonferroni correction [34]. We adopted MAGeCK, which developed α-RRA, to select the top α% sgRNAs from the ranking list. The *P* values of sgRNAs lower than a threshold (0.25 for instance) were selected. Only the top sgRNAs of one gene were considered in the RRA calculation, thus making *ρ* = min(*β*_1, *n*_, *β*_2, *n*_,  … , *β*_*j*, *n*_), in which 1 ≤ *j* ≤ *n*.

### Negative screening data analysis

During the analyzing process of positive screening at high MOI based on the iBAR strategy, we modified the model-estimated variance of sgRNAs with different fold change directions among corresponding barcodes. But for negative screening, most of the non-functional sgRNAs would be unchanged. So the variance modification algorithm based on fold change directions of corresponding barcodes becomes not sufficient to justify weather certain sgRNA is a false-positive result. Therefore, we treated barcodes as internal replicates directly. When taking iBAR into consideration, we performed two times robust rank aggregation for the negative screening rather than variance adjustment for the inconsistent sgRNAs^iBAR^. The first round of robust rank aggregation aggregates the sgRNA^iBAR^ level to sgRNA level, and the second round aggregates the sgRNA level to gene level.

### Validation of candidate genes

To validate each gene, we chose two sgRNAs designed in the library and cloned into a lentiviral vector with a puromycin selection marker. We mixed two sgRNA plasmids and co-transfected them into HEK293T cells with two lentiviral package plasmids (pVSVG and pR8.74) using the X-tremeGENE HP DNA transfection reagent (Roche). The HeLa cells stably expressing Cas9 were infected with the lentivirus for 3 days and treated with 1 μg/ml puromycin for 2 days. Then, 5000 cells were added into each well, and five replicates were obtained for each group. After 24 h, the experimental groups were treated with 150 ng/ml 6-TG, and the control groups were treated with normal medium for 7 days. Then, MTT (Amresco) staining and detection were performed following the standard protocol. The experimental wells treated with 6-TG were normalized to the wells without 6-TG treatment.

## Additional files

Additional file 1: **Figure S1.** CRISPR screening of a collection of sgRNAs^iBAR-ANTXR1^ containing all 4096 types of iBAR_6_ divided by the GC contents of iBARs. Figure S2. Evaluation of the effects of iBARs on sgRNA activity. Figure S3. DNA sequences of the designed oligos. Figure S4. The sgRNA^iBAR^ read counts for *CSPG4* targeting (a), *SPPL3* targeting (b), *UGP2* targeting (c), *KATNAL2* targeting (d), *HPRT1* targeting (e), *RNF212B* targeting (f), *SBNO2* targeting (g) and *ERAS* targeting (h) before (Ctrl) and after (Exp) TcdB screening at MOI of 10 calculated by MAGeCK in two replicates. Figure S5. sgRNA distribution and coverage in different samples. Figure S6. The Pearson Correlation of log10 (fold change) of all genes between two biological replicates after 6-TG screening at an MOI of 3. Figure S7. Mean-variance model of all the sgRNAs^iBAR^ after variance adjustment using MAGeCK^iBAR^ analysis. Figure S8. Efficiency of original designed sgRNAs targeting *MLH1*, *MSH2*, *MSH6* and *PMS2*. Figure S9. Fold changes of each sgRNA^iBAR^ targeting the indicated top candidate genes (*HPRT1*, *ITGB1*, *SRGAP2* and *AKTIP*) in two experimental replicates. Figure S10. The sgRNA^iBAR^ read counts for targeting *ITGB1* (a), *SRGAP2* (b), *AKTIP* (c), *ACTR3C* (d), *PPP1R17* (e), *ACSBG1* (f), *CALM2* (g), TCF21 (h) and *KIFAP3* (i) in two replicates. Figure S11. The sgRNA^iBAR^ read counts for targeting *GALR1* (a), *DUPD1* (b), *TECTA* (c), *OR51D1* (d), *Neg89* (e) and *Neg67* (f) in two replicates. Figure S12. Normalized sgRNA read counts of *HPRT1*, *FGF13*, *GALR1* and Neg67 via conventional MAGeCK analysis in two experimental replicates. Figure S13. Assessment of screen performance through MAGeCK and MAGeCK^iBAR^ analyses by using gold standard essential genes as determined by ROC curves. Figure S14. The effects of different lengths of iBARs on sgRNA activity. (PDF 4136 kb)

Additional file 2: **Table S1.** Results of sgRNA^iBAR-ANTXR1^ library screening for the cytotoxicity of PA/LFnDTA. Table S2. Sequences of the human genome-scale sgRNA^iBAR^ library. Table S3. Results of the human genome-scale sgRNA^iBAR^ library screening at different MOI for the cytotoxicity of TcdB using MAGeCK^iBAR^ analysis. (XLSX 15253 kb)

Additional file 3: **Table S4.** Results of the human genome-scale sgRNA^iBAR^ library screening at different MOI for the cytotoxicity of TcdB using conventional MAGeCK analysis. Table S5. Results of the human genome-scale sgRNA^iBAR^ library screening for the cytotoxicity of 6-TG using MAGeCK^iBAR^ analysis. Table S6. Results of the human genome-scale sgRNA^iBAR^ library screening for the cytotoxicity of 6-TG using conventional MAGeCK analysis. Table S7. Top 20 gene list of two biological replicates using MAGeCK^iBAR^ and MAGeCK analysis. Table S8. sgRNA design for the functional validation of candidate genes from 6-TG screening and sgRNA design for the test of iBAR effects on activity. Table S9. Primers used for PCR amplification of the genomic DNAs and for library construction. Table S10. Comparison of the numbers of cells required for CRISPR library construction for TcdB screenings at different MOIs. (XLSX 12014 kb)
