# Supplementary material for: Guide RNAs with embedded barcodes boost CRISPR-pooled screens
Source: Genome Biol. 2019 Jan 24;20:20. doi: 10.1186/s13059-019-1628-0 (PMC6345036; doi:10.1186/s13059-019-1628-0)
Supplement: Supplementary file 1 — Figure S1. CRISPR screening of a collection of sgRNAsiBAR-ANTXR1 containing all 4096 types of iBAR6 divided by the GC contents of iBARs. Figure S2. Evaluation of the effects of iBARs on sgRNA activity. Figure S3. DNA sequences of the designed oligos. Figure S4. The sgRNAiBAR read counts for CSPG4 targeting (a), SPPL3 targeting (b), UGP2 targeting (c), KATNAL2 targeting (d), HPRT1 targeting (e), RNF212B targeting (f), SBNO2 targeting (g) and ERAS targeting (h) before (Ctrl) and after (Exp) TcdB screening at MOI of 10 calculated by MAGeCK in two replicates. Figure S5. sgRNA distribution and coverage in different samples. Figure S6. The Pearson Correlation of log10 (fold change) of all genes between two biological replicates after 6-TG screening at an MOI of 3. Figure S7. Mean-variance model of all the sgRNAsiBAR after variance adjustment using MAGeCKiBAR analysis. Figure S8. Efficiency of original designed sgRNAs targeting MLH1, MSH2, MSH6 and PMS2. Figure S9. Fold changes of each sgRNAiBAR targeting the indicated top candidate genes (HPRT1, ITGB1, SRGAP2 and AKTIP) in two experimental replicates. Figure S10. The sgRNAiBAR read counts for targeting ITGB1 (a), SRGAP2 (b), AKTIP (c), ACTR3C (d), PPP1R17 (e), ACSBG1 (f), CALM2 (g), TCF21 (h) and KIFAP3 (i) in two replicates. Figure S11. The sgRNAiBAR read counts for targeting GALR1 (a), DUPD1 (b), TECTA (c), OR51D1 (d), Neg89 (e) and Neg67 (f) in two replicates. Figure S12. Normalized sgRNA read counts of HPRT1, FGF13, GALR1 and Neg67 via conventional MAGeCK analysis in two experimental replicates. Figure S13. Assessment of screen performance through MAGeCK and MAGeCKiBAR analyses by using gold standard essential genes as determined by ROC curves. Figure S14. The effects of different lengths of iBARs on sgRNA activity. (PDF 4136 kb) [file 13059_2019_1628_MOESM1_ESM.pdf]

Guide RNAs with embedded barcodes boost CRISPR-pooled screens

Shiyou Zhu, Zhongzheng Cao, Zhiheng Liu, Yuan He, Yinan Wang, Pengfei Yuan, Wei Li, Feng Tian, Ying Bao, Wensheng Wei

Biomedical Pioneering Innovation Center (BIOPIC), Beijing Advanced Innovation Center for Genomics, Peking-Tsinghua Center for Life Sciences, Peking University Genome Editing Research Center, State Key Laboratory of Protein and Plant Gene Research, School of Life Sciences, Peking University, Beijing 100871, China.

Academy for Advanced Interdisciplinary Studies, Peking University, Beijing 100871, China.

EdiGene Inc, Life Science Park, 22 KeXueYuan Road, Changping District, Beijing 102206, China.

Center for Genetic Medicine Research, Children's National Health System, Washington, DC 20010, USA.

Department of Genomics and Precision Medicine, The George Washington School of Medicine and Health Sciences, Washington, DC 20010, USA.

Correspondence should be addressed to W.W. (wswei@pku.edu.cn).

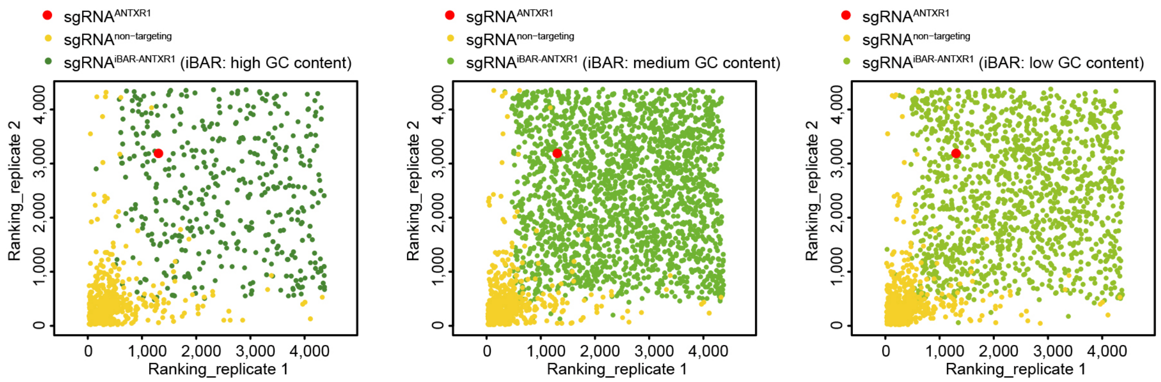

Figure S1 CRISPR screening of a collection of sgRNAs$^{iBAR\text{-}ANTXR1}$ containing all 4,096 types of $iBAR_6$ divided by the GC contents of iBARs. GC contents were categorized into three groups: high (100-66%), medium (66-33%) and low (33-0%). The rankings of two biological replicates are displayed.

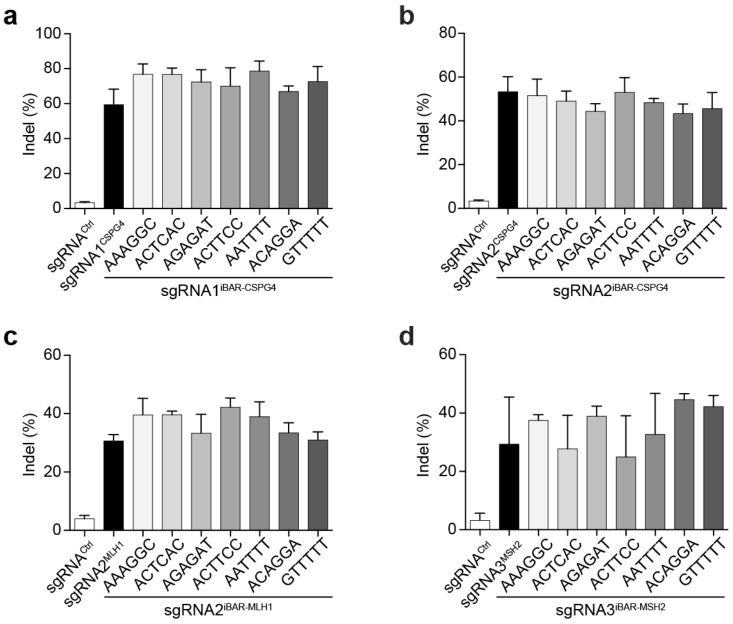

Figure S2 Evaluation of the effects of iBARs on sgRNA activity. Indels generated by sgRNA1$^{\text{iBAR-CSPG4}}$ (**a**), sgRNA2$^{\text{iBAR-CSPG4}}$ (**b**), sgRNA2$^{\text{iBAR-MLH1}}$ (**c**) and sgRNA3$^{\text{iBAR-MSH2}}$ (**d**) associated with six barcodes that appeared to be the worst in conferring cell resistance to PA/LFnDTA from the above screening as well as with GTTTTTT that was supposed to be termination signal for U6 promoter. Percentages of cleavage efficiency in the T7E1 assay were measured using Image Lab software, and data are presented as the mean ± s.d. (n = 3). All primers used are listed in Additional file 2: Table S9.

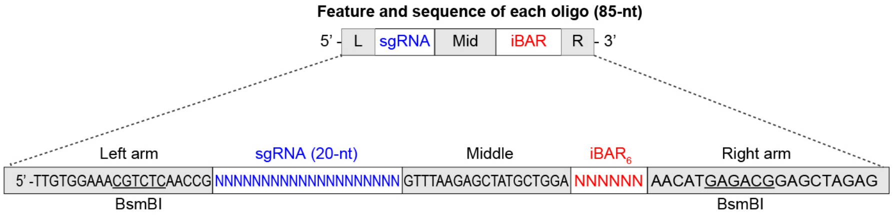

Figure S3 DNA sequences of the designed oligos. An array-synthesized 85-nt DNA oligo contains coding sequences of sgRNAs (represented in red) and barcodes (represented in blue). The left and right arms are used for primer targeting for amplification. BsmBI sites are used for cloning pooled, barcoded sgRNAs into the final expressing backbone.

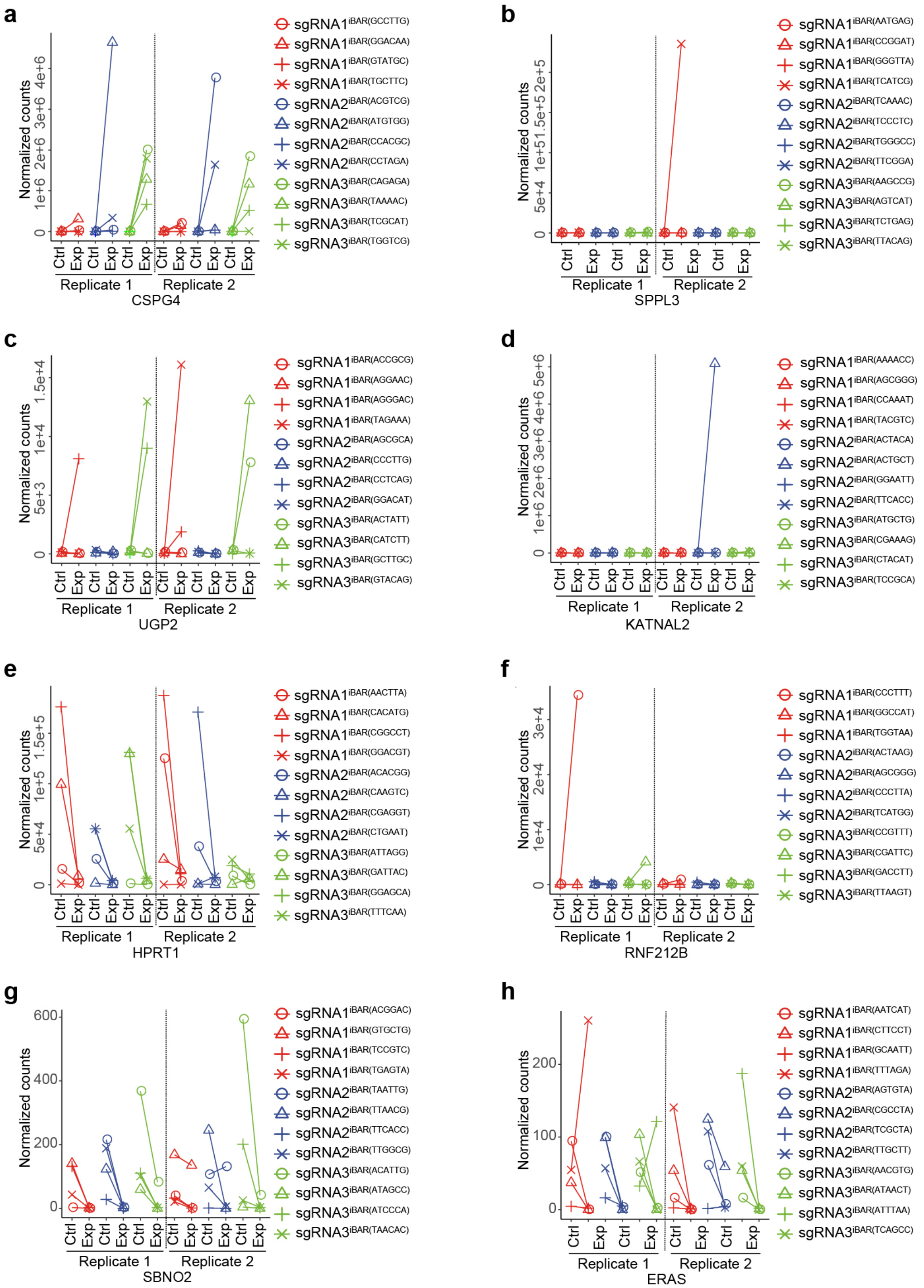

Figure S4 The sgRNA^{iBAR} read counts for *CSPG4* targeting (**a**), *SPPL3* targeting (**b**), *UGP2* targeting (**c**), *KATNAL2* targeting (**d**), *HPRT1* targeting (**e**), *RNF212B* targeting (**f**), *SBNO2* targeting (**g**) and *ERAS* targeting (**h**) before (Ctrl) and after (Exp) TcdB screening at MOI of 10 calculated by MAGeCK in two replicates.

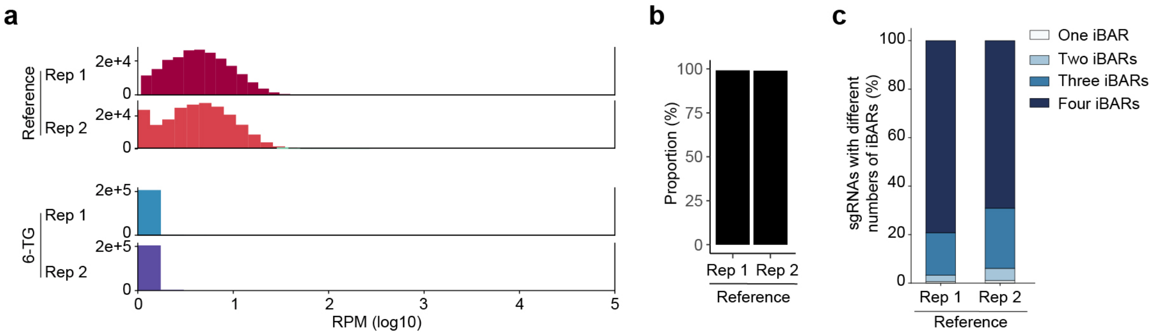

Figure S5 sgRNA distribution and coverage in different samples. (**a**) sgRNA^{iBAR} distribution of the reference and 6-TG treatment groups. The horizontal axis indicates the normalized RPM in log10, and the vertical axis indicates the number of sgRNAs. (**b**) sgRNA coverage of reference samples. The vertical axis indicates the sgRNA proportion vs. design. (**c**) Proportions of sgRNAs carrying different numbers of designed iBARs in the library.

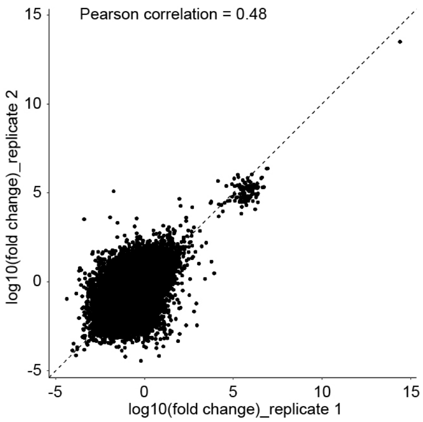

Figure S6 The Pearson Correlation of log10 (fold change) of all genes between two biological replicates after 6-TG screening at an MOI of 3.

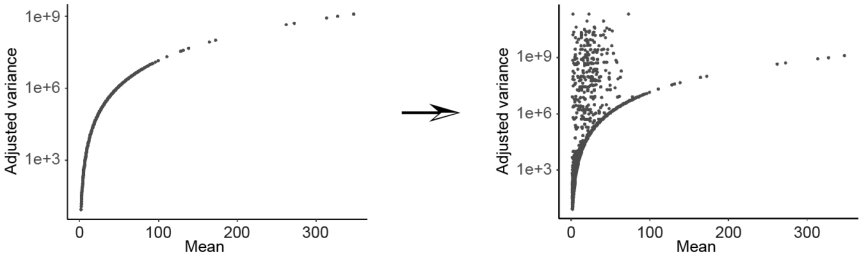

Figure S7 Mean-variance model of all the sgRNAs^{iBAR} after variance adjustment using MAGeCK^{iBAR} analysis.

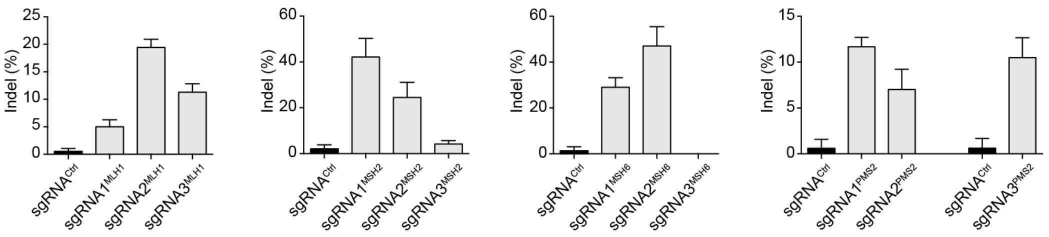

Figure S8 Efficiency of original designed sgRNAs targeting *MLH1*, *MSH2*, *MSH6* and *PMS2*. Percentages of cleavage efficiency in the T7E1 assay were measured using Image Lab software, and data are presented as the mean ± s.d. (n = 3). All primers used are listed in Additional file 2: Table S9.

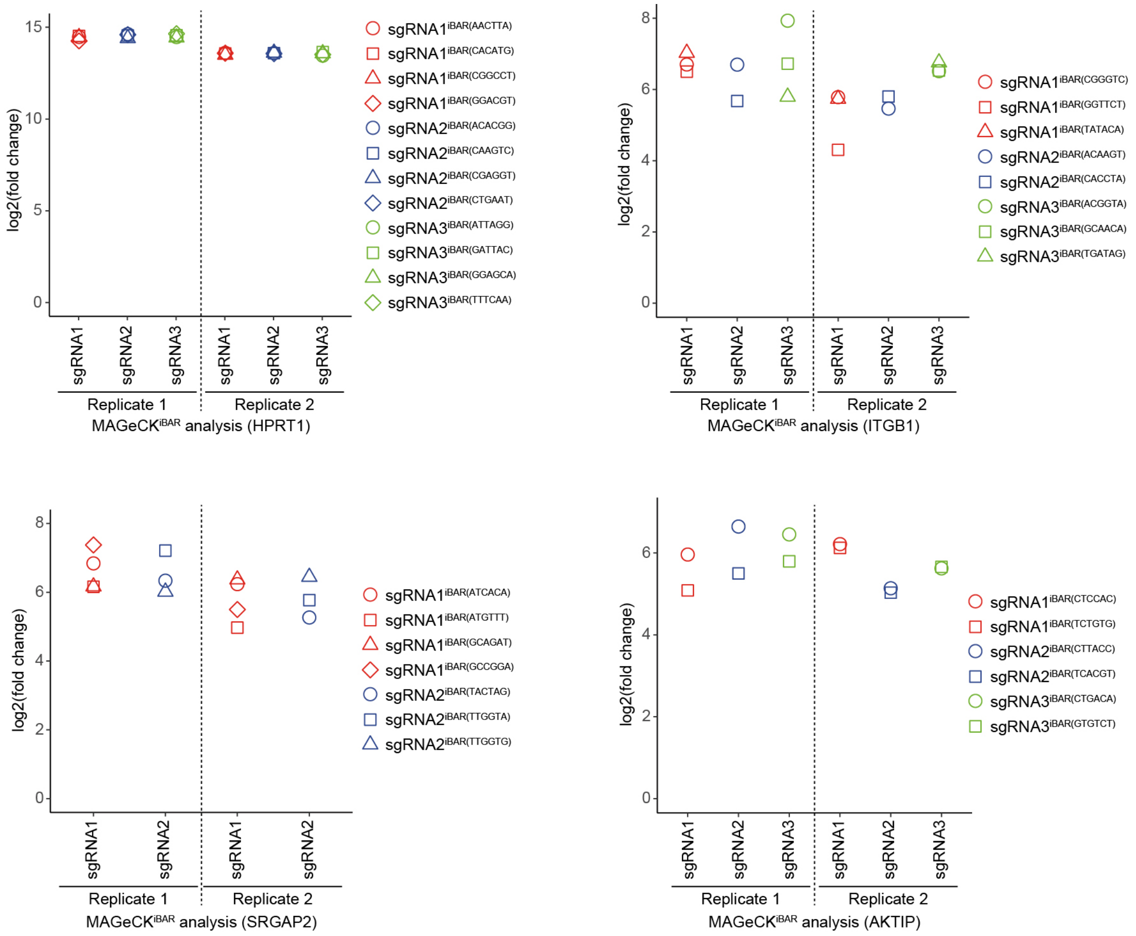

Figure S9 Fold changes of each sgRNA^{iBAR} targeting the indicated top candidate genes (*HPRT1*, *ITGB1*, *SRGAP2* and *AKTIP*) in two experimental replicates. Ctrl and Exp represent the samples before and after 6-TG treatment, respectively.

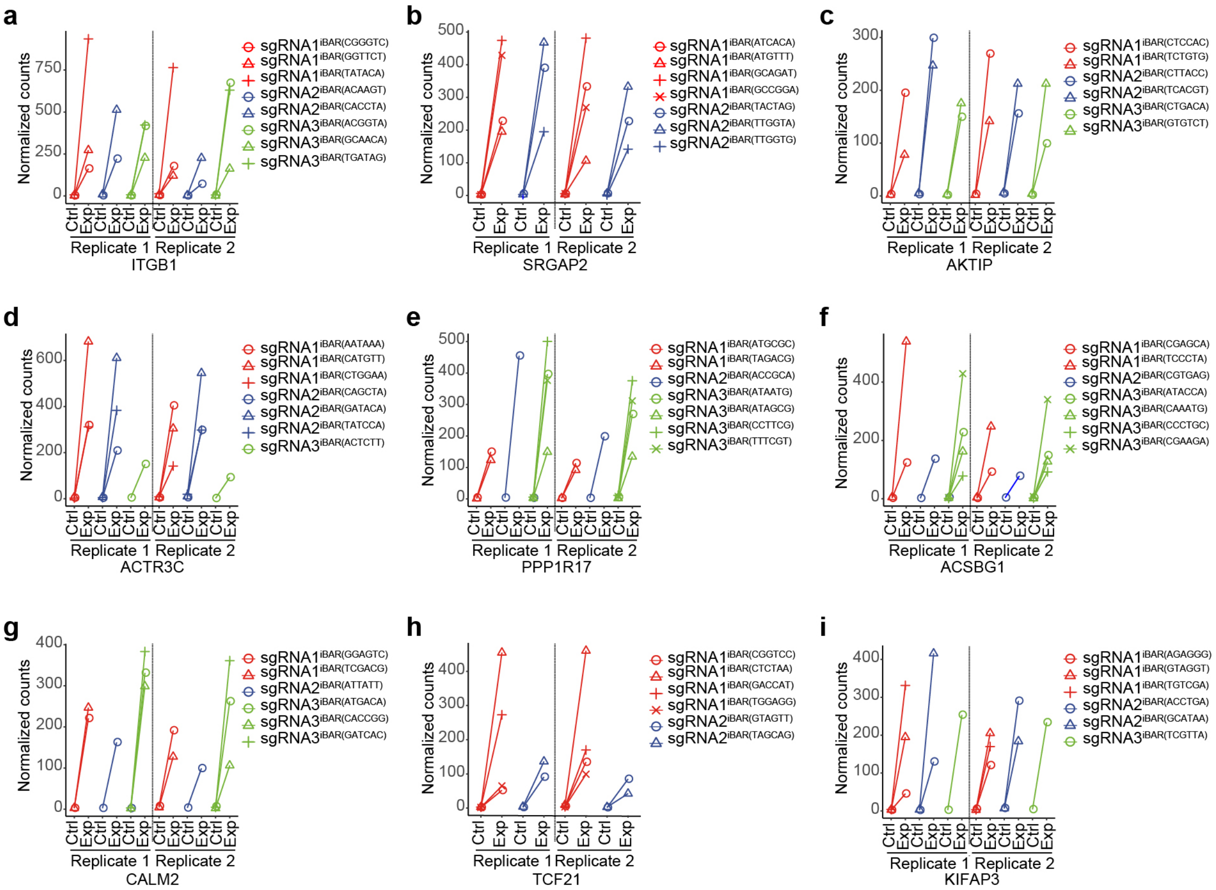

Figure S10 The sgRNA^{iBAR} read counts for targeting *ITGB1* (**a**), *SRGAP2* (**b**), *AKTIP* (**c**), *ACTR3C* (**d**), *PPP1R17* (**e**), *ACSBG1* (**f**), *CALM2* (**g**), TCF21 (**h**) and *KIFAP3* (**i**) in two replicates. Ctrl and Exp represent the samples before and after 6-TG treatment, respectively.

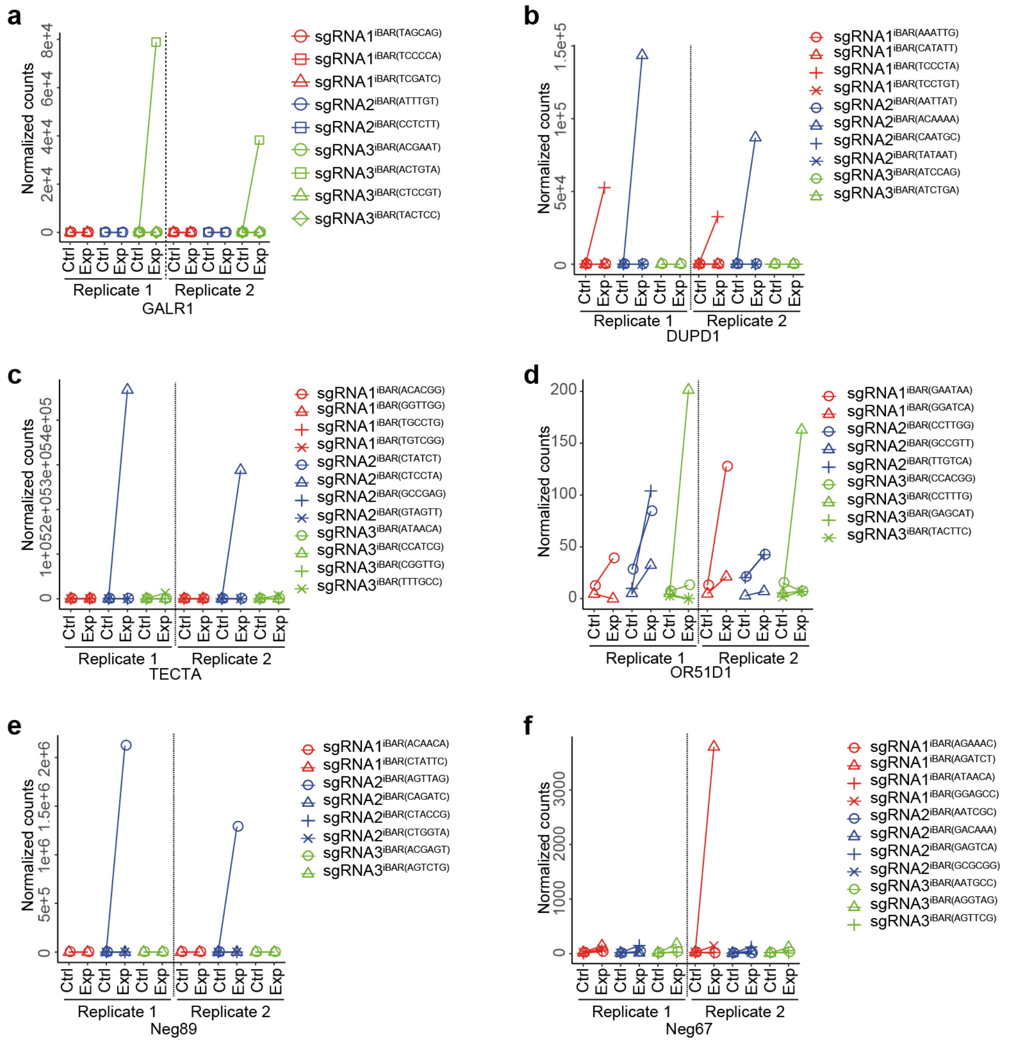

Figure S11 The sgRNA^{iBAR} read counts for targeting *GALR1* (**a**), *DUPD1* (**b**), *TECTA* (**c**), *OR51D1* (**d**), *Neg89* (**e**) and *Neg67* (**f**) in two replicates. Ctrl and Exp represent the samples before and after 6-TG treatment, respectively.

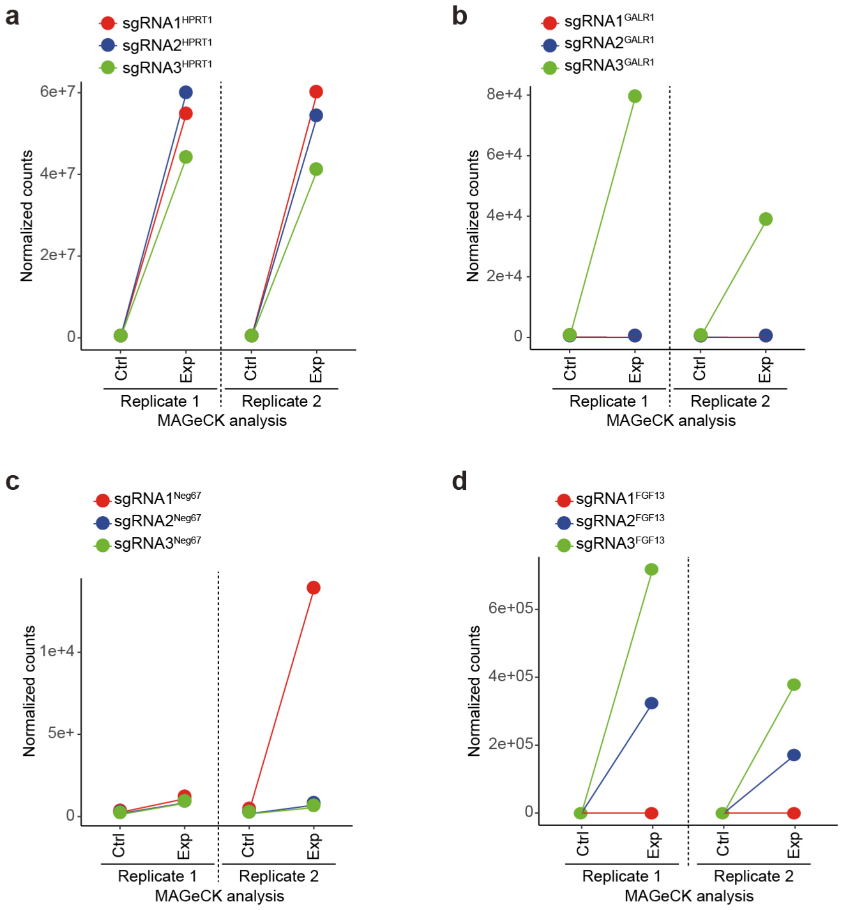

Figure S12 Normalized sgRNA read counts of *HPRT1*, *FGF13*, *GALR1* and Neg67 via conventional MAGeCK analysis in two experimental replicates. Ctrl and Exp represent the samples before and after 6-TG treatment, respectively.

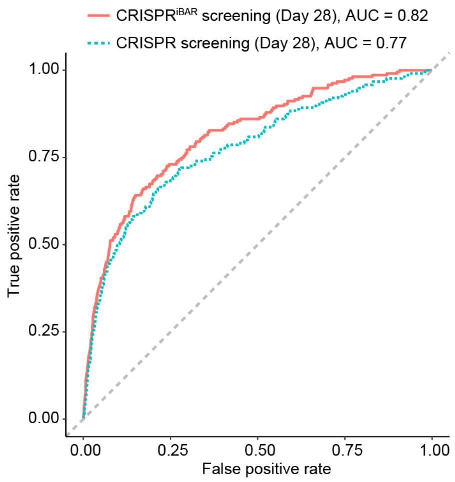

Figure S13 Assessment of screen performance through MAGeCK and MAGeCK^{iBAR} analyses by using gold standard essential genes as determined by ROC curves. The AUC (area under curve) values were shown. Dashed lines indicate the performance of a random classification model.

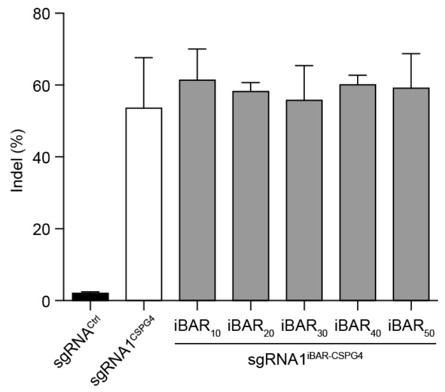

Figure S14 The effects of different lengths of iBARs on sgRNA activity. Indels were generated by sgRNA1^{CSPG4} and sgRNA1$^{iBAR\text{-}CSPG4}$ with different lengths of barcodes as indicated. Percentages of cleavage efficiency in the T7E1 assay were measured using Image Lab software, and data are presented as the mean ± s.d. (n = 3). All primers used are listed in Additional file 2: Table S9.
